# Application of POSSUM scores in post-operative complication assessment in patients undergoing general surgery: a retrospective study

**DOI:** 10.1186/s12893-025-03462-y

**Published:** 2026-01-24

**Authors:** Nan Chen, Jianning Zhai, Yuhe Zhou, Yugang Lv

**Affiliations:** 1 Key Laboratory of Carcinogenesis and Translational ResearchMinistry of Education/Beijing)Gastrointestinal Center, Unit III, Peking University Cancer Hospital & Institute, Beijing, 100142 China; 2 Bahrain Right Banner Hospital, Southern Section of Bahrain Road, Daban Town, Chifeng, 025150 China

**Keywords:** POSSUM model, Postoperative complications, General surgery, County-level hospital, Predictive ability

## Abstract

**Aims:**

This study aimed to evaluate the efficacy of the Physiological and Operative Severity Score for the Enumeration of Mortality and Morbidity (POSSUM) model in predicting postoperative complications among patients who underwent general surgery at a county-level hospital.

**Methods:**

A retrospective analysis was conducted on 401 patients admitted to the general surgery department of Bahrain Right Banner Hospital from January 2000 to April 2023. POSSUM scores were calculated based on preoperative physiological and operative severity factors. Postoperative complications were recorded and classified using the Clavien‒Dindo system.

**Results:**

The overall postoperative complication rate was 10.0%, with abdominal/pelvic infections being the most common complication (5.7%). Univariate and multivariate analyses identified sex, respiratory score, pulse score, urea nitrogen level, surgery size, and malignancy score as independent risk factors for complications. The POSSUM model demonstrated good predictive ability for postoperative complications.

**Conclusion:**

The POSSUM scoring system is a valuable tool for predicting postoperative complications in patients undergoing general surgery at county-level hospitals, enabling health care providers to identify high-risk patients and implement preventive measures. Future studies should explore the effectiveness of targeted interventions based on POSSUM scores to further improve patient outcomes.

## Introduction

General surgery is frequently associated with a myriad of complications, including infections, hemorrhages, thrombosis, impaired wound healing, and organ dysfunction [1]. These complications vary in severity, ranging from mild to life-threatening in emergency cases. The occurrence of these complications is often associated with complex surgical procedures, patients' physical conditions or anatomical variations, resulting in prolonged hospital stays and impaired quality of life. Several prediction models have been developed to predict potential risk factors, which integrate clinical and pathological factors to estimate the likelihood of postoperative complications. One of the commonly recognized prediction models, the Physiological and Operative Severity Score for the Enumeration of Mortality and Morbidity (POSSUM), has been commonly used due to its simplicity and comprehensiveness [2]. Developed by Copeland et al. in 1991, this model comprises 12 physiological factors and 6 operative severity factors to predict mortality and morbidity rates within 30 days after operation.

With the advantages of a comprehensive assessment of patient risk on the basis of readily accessible preoperative data, the POSSUM model has been validated in the general surgery departments of tertiary hospitals [3–5]. However, there are few reports regarding the application of this model in county-level secondary general hospitals. This study aimed to evaluate the efficacy of this model for the prediction of postoperative complications and risk factors in patients undergoing surgical treatment at the department of general surgery of Bahrain Right Banner Hospital in Chifeng city, Inner Mongolia.

## Methods

### Study design and participants:

This study was designed to investigate the occurrence of postoperative complications and associated factors in patients who underwent general surgery in the department of general surgery of Bahrain Right Banner Hospital from January 2000 to April 2023.

After screening was conducted by the hospital's Medical Affairs Department and Information Department, a total of 401 surgical patients admitted to the general surgery department of Bahrain Right Banner Hospital from January 2000 to April 2023 who met the inclusion and exclusion criteria were selected. The protocol was approved by the ethics committee of Bahrain Right Banner Hospital, and all participants provided written informed consent.

Inclusion Criteria: (1) Surgical patients admitted to the department of general surgery from January 2000 to April 2023; (2) Patients aged 18–85 years; (3) Surgical procedures included open or laparoscopic abdominal exploration, abdominal and pelvic hemostasis, splenectomy, partial hepatectomy, gastrointestinal perforation repair, gastric or intestinal resection, gastric or intestinal anastomosis, appendectomy, cholecystectomy (with or without choledochoscopy), and gastrointestinal tumor resection; (4) Operation performed by a group of proficient senior surgeons (these surgeons have at least 5 years of experience in abdominal surgery with more than 50 cases per year).

Exclusion Criteria: (1) Patients aged younger than 18 years or older than 85 years and (2) surgical procedures not involving abdominal organs, such as thyroid surgery, superficial mass resection, ligation, and stripping of varicose veins of the lower extremities.

## Data collection

Electronic medical records and paper medical records (including nursing notes, laboratory tests, and other medical information) were used to collect data regarding the characteristics (including surgery, pathological examination, laboratory tests, etc.) of 401 patients. POSSUM scores related to physiological factors and operative severity factors and the total score were calculated following the scoring guidelines.

Postoperative complications and the Clavien‒Dindo classification: The patients' postoperative recovery information, the types and grades of postoperative complications, and the strategies for managing these complications were accurately recorded.

## Statistical analysis

Categorical variables were analyzed using the χ2 test or Fisher’s exact test. A two-sided *p* value < 0.05 indicated statistical significance. All statistical analyses were conducted using SPSS software (Version 26.0; IBM Corp., New York, USA).

## Results

### POSSUM scores and distribution in a cohort of 401 surgical patients

**Fig. 1:**
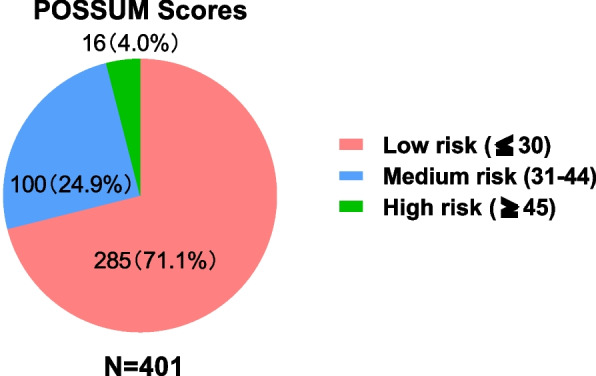
Distribution of POSSUM Scores Among Patients

The patients' clinical and pathological data were used to determine the POSSUM scores of the total population, which are summarized in Tables 1 and 2 in which the physiological and operative severity scores are presented, respectively. The physiological score of the 401 patients was 18.62, with a standard deviation of 4.76, as shown in Table 1, whereas the surgical severity score of these patients was 10.42, with a standard deviation of 1.77, as shown in Table 2. Therefore, the average score for the 401 patients was 28.89 ± 5.25. According to the scoring classification (low risk ≤ 30, medium risk 31–44, high risk ≥ 45), 285 patients (71.1%) were in the low-risk group, 100 patients (24.9%) were in the medium-risk group, and 16 patients (4.0%) were in the high-risk group. The distribution of patient risk is illustrated in Fig. 1.

**Table 1:** Summary of POSSUM Physiological Scores of Surgical Patients at Bahrain Right Banner Hospital (*N* = 401)

Factor	N (%)	Factor	N (%)
Age Score		**Hemoglobin Score**	
1 (≤ 60)	289 (72.1%)	1	233 (58.1%)
2 (61–70)	83 (20.7%)	2	118 (29.4%)
4 (≥ 71)	29 (7.2%)	4	35 (8.7%)
8	0 (0%)	8	15 (3.7%)
Sex		**White Blood Cell Score**	
Male	196 (48.9%)	1	245 (61.1%)
Female	205 (51.1%)	2	143 (35.7%)
Cardiac Score		4	13 (3.2%)
1	337 (84.0%)	8	0 (0%)
2	29 (7.2%)	**Urea Nitrogen Score**	
4	35 (8.7%)	1	343 (85.5%)
8	0 (0%)	2	47 (11.7%)
Respiratory Score		4	9 (2.2%)
1	323 (80.5%)	8	2 (0.5%)
2	11 (2.7%)	**Serum Sodium Score**	
4	66 (16.5%)	1	388 (96.8%)
8	1 (0.2%)	2	13 (3.2%)
Systolic Blood Pressure Score		4	0 (0%)
1	183 (45.6%)	8	0 (0%)
2	185 (46.1%)	**Serum Potassium Score**	
4	23 (5.7%)	1	376 (93.8%)
8	10 (2.5%)	2	21 (5.2%)
Pulse Score		4	3 (0.7%)
1	214 (53.4%)	8	1 (0.2%)
2	139 (34.7%)	**Electrocardiogram Score**	
4	36 (9.0%)	1	332 (82.8%)
8	12 (3.0%)	2	43 (10.7%)
Glasgow Coma Score		4	0 (0%)
1	401 (100.0%)	8	26 (6.5%)
2	0 (0%)	**Total Physiological Score**	18.62 ± 4.76
4	0 (0%)		
8	0 (0%)

**Table 2:** Summary of POSSUM Surgical Severity Scores of Surgical Patients at Bahrain Right Banner Hospital (*N* = 401)

Factor	N (%)
Surgery Size	
1 (Minor)	1 (0.2%)
2 (Moderate)	60 (15.0%)
4 (Major)	337 (84.0%)
8 (Extensive)	3 (0.7%)
Surgery Frequency Score	
1	401 (100.0%)
2	0 (0%)
4	0 (0%)
8	0 (0%)
Total Blood Loss Score	
1	401 (100.0%)
2	0 (0%)
4	0 (0%)
8	0 (0%)
Wound Contamination Score	
1	386 (96.3%)
2	12 (3.0%)
4	3 (0.7%)
8	0 (0%)
Malignancy Score	
1	390 (97.3%)
2	6 (1.5%)
4	5 (1.2%)
8	0 (0%)
Timing of Surgery Score	
1	208 (51.9%)
2	0 (0%)
4	193 (48.1%)
8	0 (0%)
Total Surgical Severity Score	10.42 ± 1.77

## Summary of postoperative complications

Overall, 40 of the 401 patients (10.0%) experienced postoperative complications, with 361 patients experiencing no complications. The most frequent complications were abdominal/pelvic infections (23 patients), followed by atelectasis (10 patients), urinary retention (2 patients), anastomotic leakage (2 patients), wound infections (1 patient), bleeding (1 patient), and cardiac-related complications (1 patient). According to the Clavien‒Dindo grading system, 4 patients were classified as Grade I, 29 as Grade II, 5 as Grade III, and 2 as Grade IV with no Grade V complications, as shown in Table 3.

**Table 3:** Postoperative Complications in Surgical Patients at Bahrain Right Banner Hospital (*N* = 401)

**Postoperative Complication**	N (%)
Total Complications	40 (10.0%)
Abdominal/Pelvic Infection	23 (5.7%)
Atelectasis	10 (2.5%)
Wound Infection	1 (0.2%)
Urinary Retention/Urological Infection	2 (0.5%)
Anastomotic Leakage	2 (0.5%)
Abdominal/Pelvic Bleeding	1 (0.2%)
Cardiac-Related Complications	1 (0.2%)
Others	0 (0%)
Perioperative Death (≤ 60 Days)	0 (0%)
Complication Grade (Clavien‒Dindo)	
0	361 (90.0%)
I	4 (1.0%)
II	29 (7.2%)
III	5 (1.2%)
IV	2 (0.5%)
V	0 (0%)

## Statistical Analysis of POSSUM Scores and Postoperative Complications

**Fig. 2:**
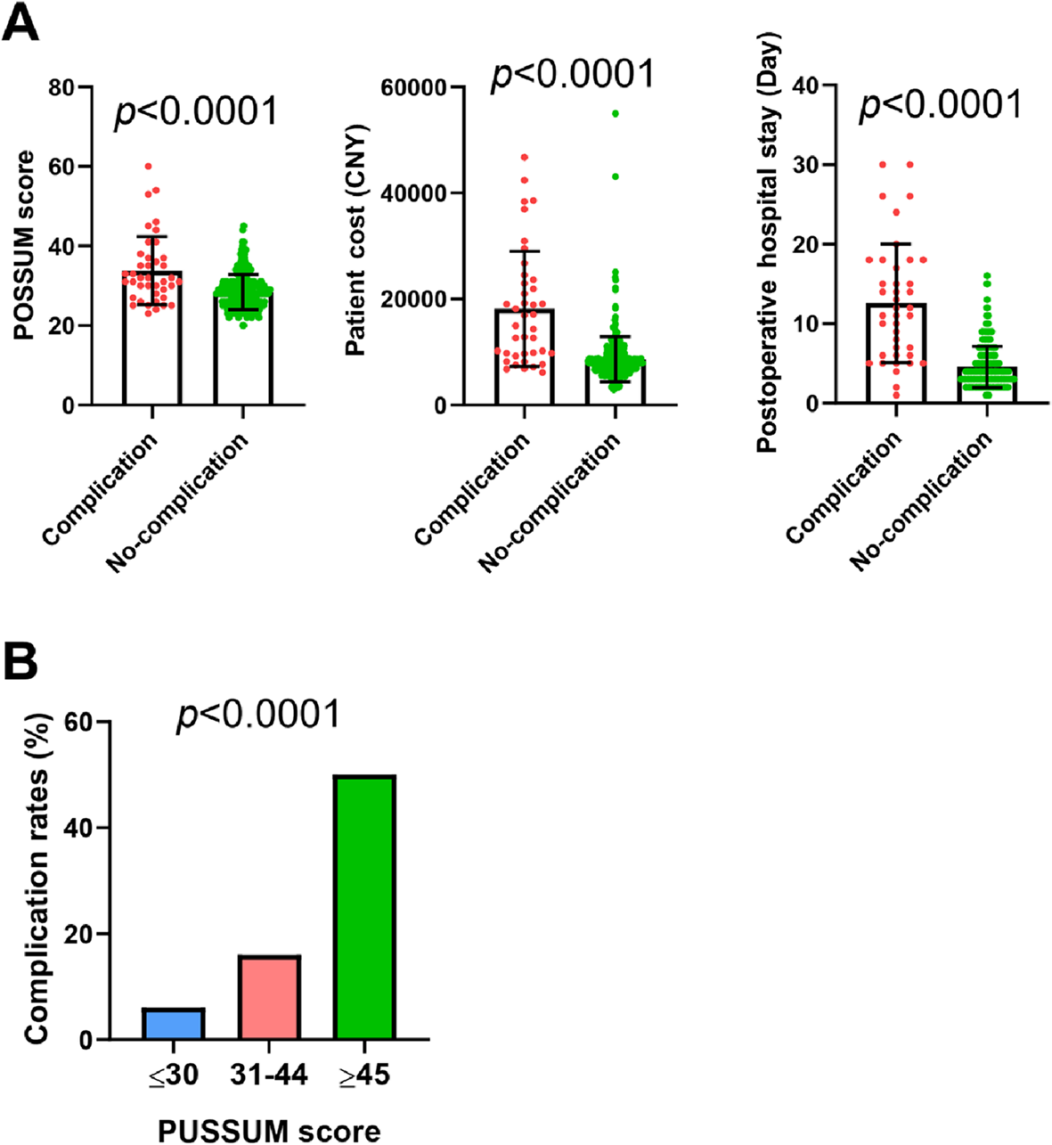
Statistical Analysis of POSSUM Scores, Postoperative Complications. **A** The correlation of complication with POSSUM score, patient costs, postoperative hospital stay. **B** Comparison of complication rates among low (≤ 30), medium (31–44), and high (≥ 45) POSSUM score groups (*p* < 0.0001)

The occurrence of postoperative complications was significantly and positively correlated with the POSSUM score (*p* < 0.0001), postoperative hospital stay (*p* < 0.0001), and total patient cost (*p* < 0.0001), as shown in Fig. 2A. As shown in Fig. 2A, the average POSSUM score for the group of patients without complications was 28.33, with a standard deviation of 4.42, whereas it was 33.83, with a standard deviation of 8.60, for the group of patients with complications. The average total cost for the no-complication group was 8650.64 Chinese yuan, with a standard deviation of 4296.8, whereas for the complication group, it was 18,197.70 (CNY), with a standard deviation of 10,873.76 (CNY).

After patients were stratified into low-, medium-, and high-risk groups (low risk ≤ 30, medium risk 31–44, high risk ≥ 45), significant differences were observed in terms of complication rates: 6% in the low-risk group, 16% in the medium-risk group, and 50% in the high-risk group, with statistically significant differences between the groups, as shown in Fig. 2B (*p* < 0.0001).

## Univariate and multivariate Analyses of the Associations Between Different Clinical Factors in the POSSUM Scoring System and Complications

Univariate analysis revealed that age (*p* = 0.003), sex (*p* = 0.007), cardiac sign score (*p* = 0.000), respiratory system score (*p* = 0.000), pulse score (*p* = 0.001), urea nitrogen score (*p* = 0.000), electrocardiogram score (*p* = 0.007), surgery size (*p* = 0.002), wound contamination score (*p* = 0.000), and malignancy score (*p* = 0.000) were significantly associated with complications (Table 4).

**Table 4:** Correlation Between Complications and POSSUM Factors in Patients Undergoing General Surgery at Bahrain Right Banner Hospital (*N* = 401)

**Age Score**			**0.003**	**Serum Sodium Score**			**0.78**
1 (≤ 60)	266 (73.7%)	23 (57.5%)		1	349 (96.7%)	39 (97.5%)	
2 (61–70)	74 (20.5%)	9 (22.5%)		2	12 (3.3%)	1 (2.5%)	
4 (≥ 71)	21 (5.8%)	8 (20.0%)		4	0 (0%)	0 (0%)	
8	0 (0%)	0 (0%)		8	0 (0%)	0 (0%)	
Sex			**0.007**	**Serum Potassium Score**			**0.829**
Male	168 (46.5%)	28 (70.0%)		1	339 (93.9%)	37 (92.5%)	
Female	293 (53.5%)	12 (30.0%)		2	18 (5.0%)	3 (5.0%)	
Cardiac Symptoms			**N/A**	4	3 (0.8%)	0 (0%)	
1	310 (85.9%)	27 (67.5%)		8	1 (0.3%)	0 (0%)	
2	20 (5.5%)	9 (22.5%)		**Electrocardiogram Score**			**0.007**
4	31 (8.6%)	4 (10.0%)		1	306 (84.8%)	26 (65.0%)	
8	0 (0%)	0 (0%)		2	34 (9.4%)	9 (22.5%)	
Respiratory Score			**N/A**	4	0 (0%)	0 (0%)	
1	299 (82.8%)	24 (60.0%)		8	21 (5.8%)	5 (12.5%)	
2	8 (2.2%)	3 (7.5%)		**Surgery Size**			**0.002**
4	54 (15.0%)	12 (30.0%)		1 (Minor)	1 (0.3%)	0 (0%)	
8	0 (0%)	1 (2.5%)		2 (Moderate)	59 (16.3%)	1 (2.5%)	
Systolic Blood Pressure Score			**0.151**	4 (Major)	300 (83.1%)	37 (92.5%)	
1	168(46.5%)	15 (37.5%)		8 (Very Major)	1 (0.3%)	2 (5.0%)	
2	165 (45.7%)	20 (50.0%)		**Surgery Frequency Score**			**N/A**
4	21 (5.8%)	2 (5.0%)		1	361 (100%)	40 (100%)	
8	7(1.9%)	3 (7.5%)		2	0 (0%)	0 (0%)	
Pulse Score			**0.001**	4	0 (0%)	0 (0%)	
1	199 (55.1%)	15 (37.5%)		8	0 (0%)	0 (0%)	
2	126 (34.9%)	13 (32.5%)		**Surgery Blood Loss**			**N/A**
4	28 (7.8%)	8 (20.0%)		1	361 (100%)	40 (100%)	
8	8 (2.2%)	4 (10.0%)		2	0 (0%)	0 (0%)	
Glasgow Coma Score			**N/A**	4	0 (0%)	0 (0%)	
1	401 (100.0%)	0 (0%)		8	0 (0%)	0 (0%)	
2	0 (0%)	0 (0%)		**Wound Contamination**			**N/A**
4	0 (0%)	0 (0%)		1 (Clean)	356 (98.6%)	30 (75.0%)	
8	0 (0%)	0 (0%)		2 (Contaminated)	4 (1.1%)	8 (20.0%)	
Hemoglobin Score			**0.059**	4 (Dirty)	1 (0.3%)	2 (5.0%)	
1	213 (59.0%)	20 (50.0%)		8	0 (0%)	0 (0%)	
2	108 (29.9%)	10 (25.0%)		**Malignancy Score**			**N/A**
4	29 (8.0%)	6 (15.0%)		1 (Benign)	359 (99.4%)	31 (77.5%)	
8	11 (3.0%)	4 (10.0%)		2 (Malignant)	2 (0.6%)	4 (10.0%)	
White Blood Cell Score			**0.257**	4	0 (0%)	5 (12.5%)	
1	225 (62.3%)	20 (50.0%)		8	0 (0%)	0 (0%)	
2	124 (34.3%)	19 (47.5%)		**Timing of Surgery Score**			**0.676**
4	12 (3.3%)	1 (2.5%)		1	186 (51.5%)	22 (55.0%)	
8	0 (0%)	0 (0%)		2	0 (0%)	0 (0%)	
Urea Nitrogen			**N/A**	4	175 (48.5%)	18 (45.9%)	
1	315 (87.3%)	28 (70.0%)		8	0 (0%)	0 (0%)	
2	40 (11.1%)	7 (17.5%)					
4	6 (1.7%)	3 (7.5%)					
8	0 (0%)	2 (5.0%)					

However, other clinical factors that are shown in Table 4**,** such as the systolic blood pressure score (*p* = 0.151), hemoglobin score (*p* = 0.059), white blood cell score (*p* = 0.257), serum sodium score (*p* = 0.780), serum potassium score (*p* = 0.829), and timing of surgery score (*p* = 0.676), were not significantly correlated with the occurrence of complications.

Moreover, multivariate analysis revealed that sex, respiratory score, pulse score, urea nitrogen, surgery size, and malignancy score were significantly correlated with complications in patients who underwent general surgery at Bahrain Right Banner Hospital (Table 5).

**Table 5:** Multivariate Analysis of Complications and POSSUM Factors in Patients Undergoing General Surgery at Bahrain Right Banner Hospital (*N* = 401)

Factor	OR	*P* value
Sex	0.271	0.005
Respiratory Score	1.636	0.001
Pulse Score	1.282	0.027
Urea Nitrogen	1.805	0.011
Surgery Size	2.018	0.041
Malignancy Score	11.151	0.005

## Comparison of Predictive Models using Receiver Operating Characteristic (ROC) curves

**Fig. 3:**
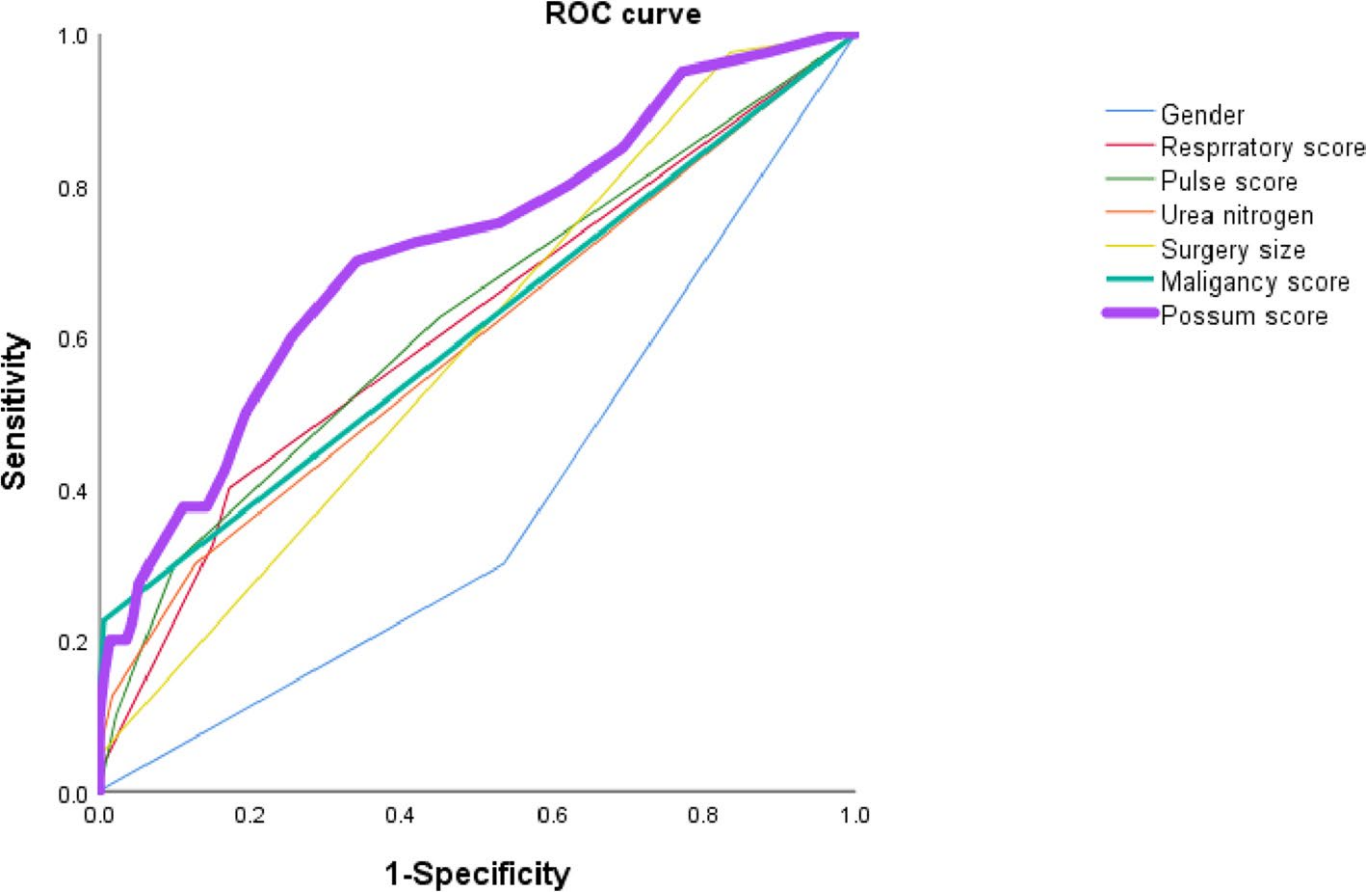
ROC curves comparing the predictive value of independent factors (sex, respiratory score, pulse score, urea nitrogen, surgery size, and malignancy score) with the overall POSSUM score

The predictive value of different factors (sex, respiratory score, pulse score, urea nitrogen, and surgery size) is shown in Fig. 3. Notably, the POSSUM score was found to be the best performing model, exhibiting the largest ROC curve area (0.7114) in the differentiation of high-risk cases compared with other factors.

## Discussion

In this study, the overall postoperative complication rate was 10.0% in the total population. Compared with the rates reported in previous studies, ranging from 10 to 25%, or even up to 37% [6], the complication rate of 10.0% reported in this study falls within a reasonable range and is slightly lower than that reported in most previous studies. This may be attributed to the variations in the distribution and complexity of tumors in general surgical procedures across hospitals of different levels. Previous studies have focused predominantly on patients from tertiary hospitals, where surgical procedures, including those involving the liver and pancreas, often involve a high proportion of Class IV surgeries, which are more highly complex. In contrast, the majority of cases in this study were Class II or III surgeries, indicating a lower level of surgical complexity. The reduced complexity of surgical procedures may be associated with decreased intricacy required for the surgical maneuvers, accounting for the relatively lower complication rate.

Regarding the incidence of complications, abdominal and pelvic infections were the leading type of complication, accounting for 5.7% of cases, followed by atelectasis, with a rate of 2.5%. Normally, maintaining unobstructed drainage and extending the duration of antibiotics were effective ways in patients with abdominal and pelvic infections. Fortunately, all patients with abdominal and pelvic infections achieved full recovery and were discharged after treatment with no fatalities. Postoperative intra-abdominal and pelvic infections remain common complications in patients undergoing general surgical procedures for several reasons. First, general surgical procedures commonly involve opening and reconstruction of the digestive tract; for example, the appendix is removed during an appendectomy. However, inflammatory exudation around the appendix is often a significant contributor to postoperative infections [7]. Additionally, compared with gastrectomy, intestinal resection and anastomosis procedures pose a greater risk of intestinal bacterial exposure during surgery. For patients with intestinal or colorectal anastomosis, especially those undergoing emergency surgeries, intra-abdominal and pelvic contamination during the operation often leads to a high proportion of intra-abdominal and pelvic infections [8]. Second, the postoperative reduction in patients' immune function, particularly in elderly patients, those with hypoproteinemia, and those with diabetes, can lead to microbial imbalance, thereby predisposing them to infections [9]. Third, inadequate postoperative drainage is also a potential cause of intra-abdominal and pelvic infections [10]. Postoperative drainage can be displaced due to factors such as movement, and excessive intra-abdominal and pelvic fluid accumulation serves as an excellent medium for the growth of bacteria or other microorganisms.

Atelectasis (2.5%) is associated with several high-risk factors, among which the baseline pulmonary function of patients is a crucial factor. In this study, 66 patients (16.5%) had a preoperative pulmonary function score of 4, indicating the presence of moderate restrictive ventilatory impairment, such as emphysema [11]. Although postoperative rehabilitative measures combine respiratory function exercises and back clapping to facilitate expectoration, factors such as surgical position (supine or lithotomy position) and patients’ low compliance with coughing and expectoration contribute to the occurrence of atelectasis. Postoperative pain and immobility, which restrict deep breathing and coughing, lead to the collapse of alveoli and the development of atelectasis.

Other postoperative complications included anastomotic leakage (0.5%), urinary retention/infection (0.5%), wound infection (0.2%, surgical site infection), bleeding (0.2%), and cardiac-related complications (0.2%). Compared with the rates of leakage reported in previous studies, the rates reported in our study were low. Considering the types of cases enrolled, only 19 patients underwent surgeries with anastomosis; the proportion of postoperative anastomotic leakage among patients who underwent anastomotic surgery was 5.3% (1/19). The low incidence of postoperative urinary tract infections is associated with postoperative urinary care. Since 2018, the general surgery department has promoted standardized postoperative management strategies for the urinary system, including perineal cleaning, bladder irrigation for high-risk patients, and patient education. Wound infection complications are often related to intraoperative contamination and high-risk factors, including diabetes. In this study, the wound infection rate was 0.2%, which may be attributed to the routine placement of wound drainage tubes or drainage strips, as well as to potential underreporting of wound infections.

In the multivariate analysis, six factors emerged as independent risk factors associated with postoperative complications: sex, respiratory score, pulse score, urea nitrogen level, surgery size, and malignancy score. Notably, the malignancy score had the highest odds ratio (OR = 11.151), indicating that surgeries for malignant tumors posed a particularly high risk for complications. Compared with nononcologic surgeries, oncologic surgeries exhibit distinct differences in terms of the extent of resection and potential lymph node dissection. Due to the aggressive and invasive nature of tumors, negative surgical margins are needed for oncologic surgeries, resulting in a greater extent of the resection. Additionally, lymph node dissection, which affects lymphatic drainage, further contributes to the potential increase in postoperative complication rates [12]. Moreover, the correlation between surgical size and complications is closely linked to the complexity of surgical procedures. For example, surgical procedures that include adhesion separation for intestinal obstruction, such as an exploratory laparotomy, may be simpler than surgeries for intestinal rupture and hemorrhage caused by trauma, with a significantly lower risk. Furthermore, in relation to complicated surgical procedures and prolonged anesthesia in patients, both surgical manipulations and anesthesia contribute significantly to an increased incidence of postoperative complications [13].

The results of this study revealed a significant correlation between the POSSUM score and the occurrence of postoperative complications, with good predictive ability, as evidenced by an area under the ROC curve (AUC) of 0.714. Although imperfect, the POSSUM is useful for identifying patients at high risk for complications, enabling health care providers at secondary hospitals to identify patients early and enact preventative measures. Building upon the findings of this study, the POSSUM has been implemented since December 2024 in the Department of General Surgery to stratify perioperative complication risks. This predictive framework particularly targets patients with preoperative high-risk factors, guiding enhanced clinical protocols. For high-risk patients, we have built up a special multidisciplinary team with intensified measures including smoking cessation protocols, homeostasis maintenance, and respiratory function training to mitigate pulmonary complications such as atelectasis supported by preoperative imaging assessments. Postoperatively, this risk-adapted approach promotes specific surveillance: frequent hematological testing, prompt antimicrobial interventions, while continuous cardiopulmonary evaluation facilitates early detection of respiratory sequelae.

However, there were a few limitations to this study. First, due to the retrospective nature of this study, the overall incidence of complications may have been underestimated on the basis of medical records, particularly during the data collection, including diagnoses, specific medication use, and length of hospital stay. Therefore, establishing a prospective complication-related registration system is very important. Second, the participants were surgical patients admitted to the department of general surgery at Bahrain Right Banner Hospital. Given the diverse range of general surgical procedures and significant differences in their complexity, the incidence of complications across various types of surgeries is confounded by multiple factors, such as patient condition and surgery size. Third, for surgeries in the general surgery departments of county-level hospitals, there were notable differences in preoperative preparation and patient status between emergency surgeries and elective surgeries. Fourth, within the current health care system, there is a misunderstanding of complications among health care providers and patients. From the health care providers' perspective, complications may be regarded as potential medical misses, whereas patients often perceive them as faults in medical care. This mutual misunderstanding regarding complications has led to the situation in which complications are underreported. This study was based on complication registration research advocated by Peking University Cancer Hospital, with the aim of enhancing data management to improve medical quality in secondary hospitals [14, 15].

This study bears notable clinical implications for practical surgical management, particularly in resource-constrained settings such as county-level general hospitals. By using the POSSUM, medical care provider can accurately stratify preoperative risk among patients, enabling personalized and intensified monitoring protocols as well as proactive intervention strategies.

## Conclusion

In conclusion, our study provides valuable insights into the utility of the POSSUM scoring system for predicting postoperative complications among general surgery patients at a county-level general hospital. Several factors, such as malignancy, surgery size, urea nitrogen, respiratory score, pulse and sex, were identified as independent parameters significantly associated with the occurrence of postoperative complications. Thus, effective monitoring and adjustment of nursing levels and intervention strategies based on the POSSUM scoring system is essential. Additional studies examining the effectiveness of targeted interventions based on the POSSUM scores are needed to demonstrate potential improvements in patient health care.

## Data Availability

The datasets used and/or analysed during the current study are available from the corresponding author on reasonable request.

## Abbreviations

POSSUM: Physiological and Operative Severity Score for the Enumeration of Mortality and Morbidity
OR: Odds radio
AUC: Area under the ROC curve
